# Respiratory Syncytial Virus–Associated Mortality Among Young Infants in Karachi, Pakistan: A Prospective Postmortem Surveillance Study

**DOI:** 10.1093/cid/ciab488

**Published:** 2021-09-02

**Authors:** Abdul Momin Kazi, Obianuju G Aguolu, Waliyah Mughis, Nazia Ahsan, Saima Jamal, Ayub Khan, Hanya M Qureshi, Inci Yildirim, Fauzia A Malik, Saad B Omer

**Affiliations:** 1 Department of Pediatrics, The Aga Khan University, Karachi, Pakistan; 2 Yale Institute for Global Health, Yale University, New Haven, Connecticut, USA; 3 Section of Infectious Diseases, Department of Medicine, Yale School of Medicine, Yale University, New Haven, Connecticut, USA; 4 Section of Infectious Diseases and Global Health, Department of Pediatrics, Yale School of Medicine, Yale University, New Haven, Connecticut, USA; 5 Department of Epidemiology of Microbial Diseases, Yale School of Public Health, Yale University, New Haven, Connecticut, USA; 6 Yale School of Nursing, Yale University, New Haven, Connecticut, USA

**Keywords:** respiratory syncytial virus, maternal vaccine, community-based mortality surveillance, verbal autopsy

## Abstract

**Background:**

Respiratory syncytial virus (RSV) is an important cause of infant morbidity and mortality and a potential target for maternal immunization strategies. However, data on the role of RSV in young infant deaths in developing countries are limited.

**Methods:**

We conducted a community-based mortality surveillance from August 2018–March 2020 for infants ≤6 months in Karachi, Pakistan. We tested (reverse transcription–polymerase chain reaction) nasopharyngeal swabs from deceased infants for presence of RSV. We performed verbal autopsies and calculated odds of RSV-associated mortality with 95% CIs and used multivariable logistic regression to evaluate associations.

**Results:**

We collected 490 nasopharyngeal specimens from 1280 eligible infant deaths. There were 377/490 (76.9%) live births and 14/377 (3.7%; 95% CI: 1.8–5.6) were RSV positive. Most deaths occurred in neonates (254/377; 67.4%), males (226/377; 59.9%), and respiratory illnesses (206/377; 54.6%). Postneonatal age (10/14, 71.4%; OR: 5.5; 95% CI: 1.7–18.0), respiratory symptoms (12/14, 85.7%; OR: 5.2; 1.2–23.7), and high RSV season (9/14, 64.3%; OR: 4.4; 1.4–13.3) were associated with RSV mortality. In multivariable logistic regression analysis, respiratory symptoms (OR: 6.6; 95% CI: 1.3–32.5), RSV seasonality (6.1; 1.8–20.4), and age (9.2; 2.6–33.1) were significant predictors of RSV-associated mortality.

**Conclusions:**

RSV has a significant mortality burden in early infancy in Karachi, Pakistan. Age, RSV seasonality, and respiratory symptoms were significant predictors of RSV-associated mortality. Our findings have implications for clinical management of young infants with cold-like symptoms, policy development, and research regarding maternal immunization against RSV during pregnancy, in resource-constrained, low-income, and vaccine-hesitant populations.

## Teaser

Respiratory syncytial virus is a potential target for maternal immunization with significant disease burden in young infants. Limited knowledge regarding RSV infant mortality in developing countries exists. We assessed the burden and determinants of RSV infant mortality in Karachi, Pakistan.

Respiratory syncytial virus (RSV) is a respiratory pathogen with an initial global burden of disease estimates indicating a moderate to high burden in developing countries, specifically among young infants [1–4]. This virus is a potential target for maternal immunization strategies to prevent disease and early death in young infants. However, due to a lack of definitive evidence, the role of RSV in neonatal and young infant mortality in low- and middle-income countries is not known [5]. Moreover, most current studies estimate RSV disease burden only in terms of facility-based (hospital or clinic) deaths and hospital-acquired infections. Thus, there is a knowledge gap regarding the proportion of community-based deaths due to RSV. Furthermore, there are many challenges to generating high-quality evidence for the role of RSV in infant mortality, such as lack of resources for hospital and community surveillance and diagnostics and difficulty in obtaining specimens in populations where burial occurs within hours after death [6]. Therefore, there are limited direct data on the incidence and severity of RSV and the associated early deaths in specific populations [1, 3], resulting in insufficient evidence to inform policy considerations for maternal and childhood immunization schedules [7].

Pakistan has one of the highest infant mortality rates globally due to multiple causes, but little is known about the RSV burden in infant mortality [8]. The primary objective of this study was to assess and analyze the burden and determinants of RSV mortality in infants in the urban areas of Karachi, Pakistan. We conducted a community-based mortality surveillance study to obtain more accurate estimates of RSV-associated deaths in young infants in these areas. This is a seminal community-based RSV mortality study in Pakistan, made possible due to close liaison with the communities to collect nasopharyngeal swab samples from deceased infants.

## METHODS

This was an observational community-based mortality surveillance study conducted in 4 peri-urban low-income settlements (Rehri Goth, Ibrahim Hyderi, Bhains Colony, and Ali Akbar Shah) in Karachi, Pakistan. The overall population of the catchment area is approximately 249 128, with a cohort of approximately 7525 new births per year [9].

Data were collected from August 2018 to March 2020. The primary outcome measure was prevalence of laboratory-confirmed (reverse transcription–polymerase chain reaction [RT-PCR]) RSV infection in nasopharyngeal specimens from recently deceased infants younger than 6 months. The study enrollment inclusion criteria comprised stillborn or recently deceased infants under 6 months of age in the catchment areas. Parents or other caregivers gave informed verbal consent for a nasopharyngeal swab collection from their deceased infant at the household preferably before ritual cleansing and shrouding, and before burial.

Prior to the onset of the surveillance phase, we conducted a formative phase where we explored the acceptance of study procedures within the communities and assessed the benefits to families of deceased infants for participating in the study. We obtained approval and religious rulings from religious scholars that collecting specimens via nasopharyngeal swabs after death and before burial is religiously acceptable in the context of the Islamic, Christian, and Hindu faiths. Infant death alerts were conveyed to the team from key community stakeholders, such as religious leaders, traditional birthing attendants, and graveyard personnel.

Upon receiving a death alert from a key community partner or community member, the RSV study team mobilized from the primary health center at the field site to the identified household of the deceased infant. Due to the religious beliefs of predominantly Muslim communities, burial of the deceased occurs as soon as possible. Hence, there was a short time window for enrollment before the ritual bathing, shrouding, and burial of the infant. A 24 × 7 surveillance system was established to mobilize the study team to approach a household within 25 minutes of receiving a death alert.

As Muslim burials generally occur within 24 hours of death and the staff received most alerts within 1 hour of the infant’s death, we had no concerns about the viability of specimens for collection. The nurse performing the nasopharyngeal specimen collection donned a surgical mask and clean gloves to minimize exposure. Laboratory staff were trained in specimen testing methods for RSV. After obtaining verbal consent from the deceased infant’s parents/caregivers and completing the Case Record Form (see Supplementary Material), trained personnel collected a nasopharyngeal swab from each nostril using Pernasal Dacron swabs. The swab was inserted nasally and advanced along the floor of the nose until it reached the nasopharynx and held against the posterior nasopharynx for a few seconds, based on Centers for Disease Control and Prevention (CDC) guidelines [10], before removal. The first swab was immediately placed in a Universal Transport Media (UTM) tube, then placed on cold packs or in wet ice (~2–4°C) to prevent loss in viral titer. The second swab was immediately placed in semi-solid Regan-Lowe transport medium, then placed on cold packs or in wet ice (~2–4°C) to prevent loss in bacterial titer (for a separate pertussis study not included in this manuscript). The samples were stored in UTM tubes and transported to the laboratory. Transport to the Aga Khan University Infectious Disease Research Laboratory occurred 3 times a day: morning, afternoon, and evening. During transport, the sample was held at 2–4°C. It was stored at −70°C or lower upon receipt.

### Storage and Archiving

Nasopharyngeal specimens were stored at 70°C or lower prior to nucleic acid extraction until sufficient samples were available for batch testing. Nucleic acid was extracted from frozen aliquots using QIAamp Viral RNA Mini Kit (Qiagen).

### Laboratory Tests for Primary Confirmation of Respiratory Syncytial Virus

The extracted nucleic acids (from a Roche MagNApure compact automated system) were used for 1-step real-time PCR for RSV detection, using a Qiagen 1-step RT-PCR kit. Real-time RT-PCR assays were performed with primers and probes for RSV, using the CDC protocol [11]. All assays were run with positive controls (RSV-RNA), negative controls (nuclease-free water), and internal positive controls (RNAseP) for quality-control purposes.

### Home Visits and Cause of Death Consultation

A psychologist trained the field team in grief support and communication skills to conduct home visits for bereaved parents at the time of and up to 2 months after the infant’s death. The World Health Organization (WHO) verbal and social autopsy tool was administered during grief-support home visits within a week of enrollment. A cause of death report containing verbal and social autopsy findings and laboratory-confirmed PCR results for RSV was shared with enrolled parents alongside continuous community engagement activities.

### Verbal Autopsy—Identifying Cases and Controls

To determine the cause of death, the following was considered: (1) nasopharyngeal swab test results, (2) medical records of symptoms (when available), and (3) verbal and social autopsy. Symptoms obtained through verbal autopsy as well as medical records, when available, were considered to determine the likely cause of death. Cases were deceased infants who had respiratory symptoms as per acute lower respiratory infection (ALRI) criteria (cough, wheezing, rhonchus, crackles, etc). Controls were deceased infants who did not have any of the defined respiratory symptoms.

### Statistical Analysis

To determine RSV burden, we calculated crude RSV mortality proportions and the odds of having laboratory-confirmed RSV among cases and controls of deceased infants captured through our surveillance system. Our symptom-based case definitions for respiratory disease cases was a deceased live-birth infant who had at least 1 of these respiratory symptoms that occurred within 2 weeks immediately preceding death, with no asymptomatic period between symptom onset and death: (1) cough, (2) difficulty breathing, (3) chest wall indrawing, (4) wheezing, (5) rhonchus, or (6) crackles when breathing and any one of the very severe symptoms (head-nodding, lethargy, convulsions, cyanosis, difficulty breastfeeding, or vomiting) [9, 12]. Very severe ALRI includes presence of respiratory symptoms plus unconsciousness, poor feeding, lethargy, and convulsions. Controls were defined as deceased live-birth infants who did not meet the symptom-based case definition above.

To determine predictors of RSV mortality, we compared the distribution of covariates such as hospital deaths versus community deaths and gender, age, or seasonality among infants with and without laboratory-confirmed RSV (controls) using chi-square tests and odds ratios (ORs). Since we did not have access to the medical records of most of the deceased infants to determine the details of length of hospital stay for the entire sample, we defined hospital deaths as deaths that occurred inside a tertiary care hospital, regardless of duration of time in the hospital. Community deaths were defined as infants who died either on the way to the hospital, in the traditional birth attendant’s home, at the clinic, at home, or in a community-based health center. Age was categorized as 0–3 months and 4–6 months, as well as neonatal (0–28 days old) and post-neonatal (29 days and older) for analysis. Stillborn infants were not included in the analyses as we would not expect to see any RSV in them (never exposed to RSV). RSV seasonality in Pakistan was defined based on a prospective surveillance study conducted at the Aga Khan University Hospital in Karachi, Pakistan [13]. RSV is identified year-round in Pakistan but is most prevalent during the rainy season, which is from August to October with a peak in September. For analyses, we classified RSV seasonality as “high” (August to October) and “low” (November to July). Multivariable logistic regression was used to assess the association of these covariates on RSV positivity among deceased infants. This will provide key information regarding the extent to which severe disease (eg, requiring care in a hospital prior to death) is associated with RSV positivity, while adjusting for the presence or absence of other covariates.

Approval from the Ethical Review Committee/Institutional Review Boards (IRBs) of Yale University and Aga Khan University was obtained. Study protocol, all data-collection instruments, and consent forms (into English as well as in local languages) were also approved by both institutions’ IRBs.

## RESULTS

**Figure 1.**
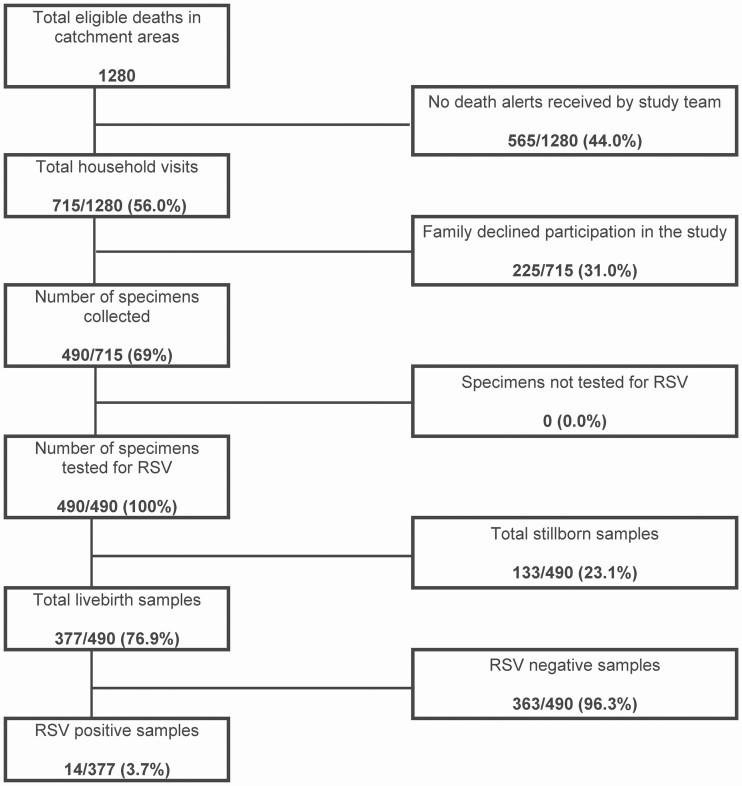
Cohort diagram on infant mortality and data collected and tested for RSV from August 2018 to March 2020 in Karachi, Pakistan. Inclusion criteria were deceased infants aged less than 6 months in catchment areas. Patients were excluded if they were older than 6 months or from out of catchment areas. Abbreviation: RSV, respiratory syncytial virus.

**Figure 2.**
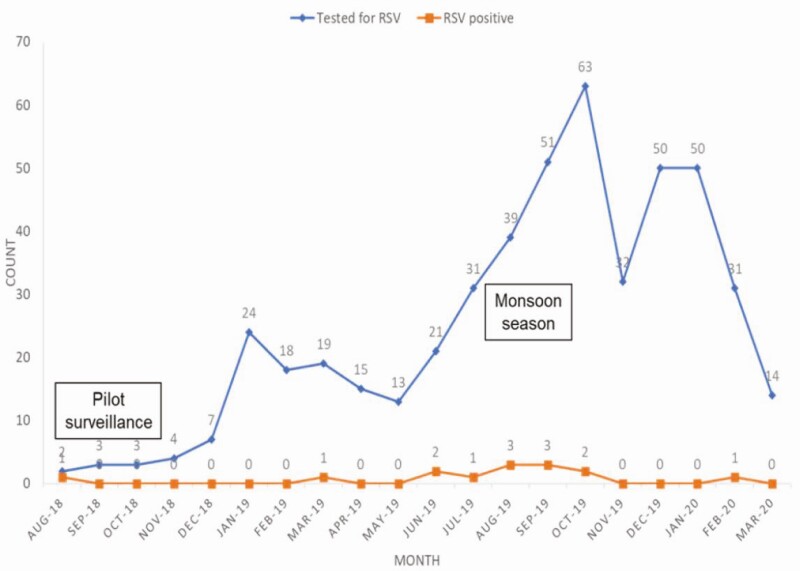
Number of deceased infants enrolled and RSV-positive cases by month and year. This shows trends in enrollment between August 2018 and March 2020 with number of RSV-positive cases per month. August 2018 to December 2018 was the pilot surveillance phase. The monsoon season (lots of rain) in Karachi, Pakistan, is between July and September. This coincides with their high-RSV season, which is from August to October. Abbreviation: RSV, respiratory syncytial virus.

Of 1280 eligible infant deaths that occurred between August 2018 and March 2020 in the catchment areas, 490 nasopharyngeal specimens were obtained; 113 of 490 (23.1%) were stillborn and 14 of 377 (3.7%; 95% confidence interval [CI]: 1.8–5.6%) live-birth infant specimens tested positive for RSV. Figure 1 shows the cohort diagram. Most of the deceased live-birth infants were males (226/377; 59.9%) and aged 0–3 months old (332/377; 88.1%) or neonates (254/377; 67.4%). The mean age of the population studied was 30.3 months (95% CI: 28.7–31.9 months) and the median age was 6.0 months. Figure 2 indicates trends in enrollment between August 2018 and March 2020 with RSV seasonality.

### Attribution of Respiratory Deaths

The proportion of deceased infants with laboratory-confirmed RSV was 14 of 377 (3.7%) (Table 1). There was a similar proportion of deaths in the community or hospital; however, most of the RSV cases (10/14; 71.4%) died in a hospital. It is important to note that, for some of the infants whose medical records were available, while they did not spend up to 24 to 48 hours in the hospital where they died, they presented at several hospitals for the fatal illness. Two of the RSV-positive cases who died in the hospital within 24 hours of hospitalization were admitted to the intensive care unit prior to death.

**Table 1.** Clinical and Demographic Characteristics of the Deceased Live-Birth Infants: August 2018–March 2020, Karachi, Pakistan

	RSV Status		
Variable	Negative, n (%)	Positive, n (%)	Total, n (%)
Total, n (%)	363 (96.3)	14 (3.7)	377 (100.0)
Age at death			
0–3 months	320 (88.2)	12 (85.7)	332 (88.1)
≥3 months	43 (11.8)	2 (14.3)	45 (11.9)
Age at death			
Neonatal: 0–28 days	250 (68.9)	4 (28.6)	254 (67.4)
Non-neonatal: ≥29 days	113 (31.1)	10 (71.4)	123 (32.6)
Sex			
Female	146 (40.2)	5 (35.7)	151 (40.1)
Male	217 (59.8)	9 (64.3)	226 (59.9)
Study site			
Ali Akbar Shah Goth	102 (28.1)	10 (71.4)	112 (29.7)
Bhains Colony	107 (29.5)	2 (14.3)	109 (28.9)
Ibrahim Hyderi	73 (20.1)	2 (14.3)	75 (19.9)
Rehri Goth	81 (22.3)	0 (0.0)	81 (21.5)
Place of death			
Community	185 (51.0)	4 (28.6)	189 (50.1)
Hospital	178 (49.0)	10 (71.4)	188 (49.9)
Symptom-based case definitions			
Case	194 (53.4)	12 (85.7)	206 (54.6)
Control	169 (46.6)	2 (14.3)	171 (45.4)
Season of illness and death			
Low (November to July)	257 (70.8)	5 (35.7)	262 (69.5)
High (August to October)	106 (29.2)	9 (64.3)	115 (30.5)
ALRI classification^a^			
ALRI	84 (23.1)	4 (28.6)	88 (23.3)
Severe ALRI	19 (5.2)	3 (21.4)	22 (5.8)
Very severe ALRI	91 (25.1)	5 (35.7)	96 (25.5)
No respiratory symptoms	169 (46.6)	2 (14.3)	171 (45.4)

Abbreviations: ALRI, acute lower respiratory infection; RSV, respiratory syncytial virus.

^a^ALRI, cough or difficulty breathing with increased respiratory rate for age; Severe ALRI, ALRI with wheeze and chest wall indrawing; Very severe ALRI, ALRI with at least 1 danger sign (eg, cyanosis, difficulty in breastfeeding or drinking, vomiting, convulsions, lethargy, unconsciousness, poor feeding, unconsciousness).

To compare the distribution of covariates among deceased infants with laboratory-confirmed RSV status vs no RSV, we calculated odds ratio (see Table 2). While the majority (67.4%; 254/377) of the deceased live-birth infants died during the neonatal period, most of the RSV-positive cases (71.4%; 10/14) were among infants who died after the neonatal period (OR: 5.5; 95% CI: 1.7–18.01). Deceased infants who had respiratory symptoms during fatal illness were more likely to test positive for RSV versus those who had no respiratory symptoms during fatal illness (12/14, 85.7%; OR: 5.2; 95% CI: 1.2–23.7). Infants who died during the high-RSV season were more likely to test positive for RSV compared with those who died during the low-RSV season (64.3%, 9/14; OR: 4.4; 95% CI: 1.4–13.3).

**Table 2.** Comparing Distribution of Covariates Among Deceased Infants with Laboratory-Confirmed Respiratory Syncytial Virus Status (Chi-Square)

Variable	Number of Observations	OR	95% Confidence Limits	*P*
Symptom-based case definitions for respiratory disease (case vs control)	377	5.2	1.2–23.7	.02
Place of death (hospital vs community)	377	2.6	.8–8.4	.10
Gender (male vs female)	377	1.2	.4–3.7	.74
Age (0–3 months vs ≥3 months)	377	1.2	.3–5.7	.78
Age (non-neonatal vs neonatal)	377	5.5	1.7–18.01	.0016
Seasonality (high vs low)	377	4.4	1.4–13.3	.0051

Number of observations shows the total number of enrolled live-birth infants tested for RSV = 377. The outcome is RSV-positive status. Abbreviations: OR, odds ratio; RSV, respiratory syncytial virus.

In multivariable logistic regression, we found that age (>28 days) (OR: 9.2; 95% CI: 2.6–33.1), having respiratory symptoms (OR: 6.6; 95% CI: 1.3–32.5), and RSV seasonality (OR: 6.1; 95% CI: 1.8–20.4) were significant predictors of testing RSV positive (Table 3).

**Table 3.** Multivariable Logistic Regression Model of Prediction of Respiratory Syncytial Virus Positivity in Infants Who Died from August 2018 to March 2020 in Karachi, Pakistan

Variables	Estimate	Standard Error	Wald Chi-Square	Pr > ChiSq	OR	95% CI
Intercept	–9.9	2.0	24.6	<.0001		
Age (non-neonate vs neonate)	2.2	0.7	11.6	.0007	9.2	2.6–33.1
Sex (male vs female)	0.3	0.6	0.2	.6255	1.4	.4–4.5
Symptom-based case definitions for respiratory disease (case vs control)	1.9	0.8	5.4	.0203	6.6	1.3–32.5
Place of death (hospital vs community)	1.0	0.6	2.5	.1167	2.7	.8–9.5
Season (high vs low)	1.8	0.6	8.6	.0034	6.1	1.8–20.4

The number of enrolled live birth infants tested for RSV = 377, chi-square = 28.9, df = 5, *P* = .0001. Probability model shows RSV status = positive; convergence criterion (GCONV = 1E-8) satisfied. References: Age: 0–3 months; Sex: female; Cases: acute lower respiratory infection; Place of death: community; and Seasonality: low. Abbreviations: CI, confidence interval; OR, odds ratio; RSV, respiratory syncytial virus.

## Discussion

This is the first community-based study examining RSV mortality in young, vulnerable infants in Pakistan. This is a seminal community-based, real-time surveillance study documenting a moderate to high burden of RSV mortality in young infants. We found the burden of infant mortality attributed to RSV in Karachi, Pakistan, to be similar to the systematic review findings by Nair et al [3] of 2.1% (1.6–2.2%) for children aged 1 year or younger in developing countries or 2.2% (1.8–2.7%) for children aged 0–5 months in developing countries by Shi et al [1]. This indicates a need for development of vaccines for RSV. Most deaths occurred among infants aged 28 days and older or 0–3 months old. This finding is important because maternally acquired RSV antibodies at birth predict protection from RSV in infants in the first 3–6 months of an infant’s life [14]. This has implications for maternal immunization against RSV during pregnancy [15]. Furthermore, our findings are in line with studies indicating that RSV incidence and mortality proportion increase during the RSV high season [16]. Moreover, the majority of the RSV-positive cases had respiratory symptoms during fatal illness. This is especially important considering that many respiratory infections such as influenza, adenovirus, or rhinovirus often present with similar (cold-like) symptoms. In young infants, RSV infections develop into serious lower respiratory tract diseases (bronchiolitis and pneumonia) with long-term sequelae (wheeze, cough, asthma, pulmonary dysfunction, bronchial reactivity, and acute respiratory disease), which may require oxygen, intubation, and/or mechanical ventilation, or worse, death [17]. This has implications for the clinical management of young infants with cold-like symptoms. During high-RSV season, there should be a high index of suspicion for RSV.

Symptom-based case definition of respiratory disease and community surveillance allowed us to collect data on infants who had never been to a hospital and therefore never identified as a case, as well as infants who may have had misclassified hospital outcomes or comorbid conditions that overshadowed the RSV diagnosis (such as congenital malformations). Community engagement and support from religious and community leaders and community religious buy-in through supporting religious rulings allowed us to conduct 24/7 surveillance and obtain consent for specimen collection in a timely, sensitive manner. In such low-income communities, home/community-acquired RSV infection may never be tested or treated in a hospital. Therefore, hospital-based data for RSV infection and mortality may not be entirely representative of the true prevalence. In this study, 4 of the 14 RSV-positive cases did not seek care in a hospital for their fatal illness.

Limitations include the unavailability of access to medical records for some of the hospitalized deceased infants. Approaching households and obtaining consent in high-refusal areas was a data-collection challenge that was overcome with community-based study advocacy strategies. We collected data over multiple high seasons (2018–2020); however, comparisons could not be made between the 3 years since the first high season (2018) was a pilot phase with few enrollments and the third high season (2020) was interrupted by the coronavirus disease 2019 (COVID-19) pandemic. The 2019 high season captured the majority of total enrollments (deaths and cases), indicating that overall mortality increases during the RSV high season.

Ongoing surveillance of the community-based RSV infant mortality study includes minimally invasive tissue sampling of lung and thorax specimens to further improve classification of upper respiratory tract infections, including RSV. As neonates and young infants have been found to be particularly vulnerable, this study has substantive implications for policy development and research regarding maternal immunization against RSV, specifically in resource-constrained, low-income, and vaccine-hesitant populations.

## Supplementary Data

Supplementary materials are available at *Clinical Infectious Diseases* online. Consisting of data provided by the authors to benefit the reader, the posted materials are not copyedited and are the sole responsibility of the authors, so questions or comments should be addressed to the corresponding author.

ciab488_suppl_Supplementary_Material

## Notes

***Acknowledgments.*** The authors thank the Aga Khan University Infectious Disease Research Laboratory and RSV study team. They also thank Dr Jeanette Guarner for the contributions to the initial design of the study and feedback on a draft of the manuscript.

***Supplement sponsorship.*** This supplement is sponsored by the Bill and Melinda Gates Foundation.

***Financial support.*** This work was supported by the Bill & Melinda Gates Foundation (grant number INV-003373).

***Potential conflicts of interest.*** I. Y. reports personal fees from Merck outside the submitted work. All other authors report no potential conflicts. All authors have submitted the ICMJE Form for Disclosure of Potential Conflicts of Interest. Conflicts that the editors consider relevant to the content of the manuscript have been disclosed.
