# Supplementary material for: Respiratory Syncytial Virus–Associated Mortality Among Young Infants in Karachi, Pakistan: A Prospective Postmortem Surveillance Study
Source: Clin Infect Dis. 2021 Sep 2;73(Suppl 3):S203–9. doi: 10.1093/cid/ciab488 (PMC8411247; doi:10.1093/cid/ciab488)
Supplement: ciab488_suppl_Supplementary_Material [file ciab488_suppl_supplementary_material.docx]

**Supplementary materials**

**Tools & Materials**

Case record forms for field site specimen collection and laboratory processing

| **COMMUNITY BASED RESPIRATORY SYNCYTIAL VIRUS MORTALITY STUDY- SURVEILLANCE PHASE** |
| --- |
| **CRF 1: CHILD IDENTIFICATION** |
|  |

| DSS ID | // | |
| --- | --- | --- |
| Interviewer ID |  |  |
| Today’s Date *(dd/mm/yyyy)* | | // |
| Infant’s Date of Birth *(dd/mm/yyyy)* | | // |
| Infant’s Date of Death *(dd/mm/yyyy)* | | // |
| Estimated time of death (hh:mm, 24 hr clock) | | \|___\|___\|:\|___\|___\| |
| Study Site | Rehri Goth  Ibrahim Hyderi  Ali Akbar Shah  Bhains Colony  NICH | |

| **Section 1: IDENTIFYING INFORMATION** | | |
| --- | --- | --- |
| **1.** | Mother’s Name |  |
| **2.** | Father’s Name |  |
| **3.** | Head of Household’s Name |  |
| **4.** | Name of the Child |  |
| **5.** | Age of the Child | Months  Days  Hours |
| **6.** | Gender of Participant | Male =1 Female =2 |
| **7.** | Consented | Yes  No  If no, please specify the reason:  If No, then after reason skip to end |
| **`8.** | Study ID | RSV- |

| **Section 2.1: NASOPHARYNGEAL SWAB COLLECTION INFORMATION** | | | | |
| --- | --- | --- | --- | --- |
| **1.** | Nasopharyngeal swab collected from right nostril | | **1** | Yes  UTM |
|  |  |  | **2** | No 🡪 Skip to 1.1 |
|  | 1.1 | If no, please specify the reason:________________________________________________ skip to section 2.2 | | |
| **2.** | Sample Collection time *(hh:mm, 24 hr clock)* | | **\|___\|___\|: ___\|___\|** | |
| **3.** | Swab taken before the ritual cleaning of the right nostril | | Yes  No | |
| **4.** | Sample Storage Condition | | **Cold Packs**   **Wet Ice** | |
| **5.** | Temperature of Coleman at the time of receiving | | - degree C | |

| **Section 2.2: NASOPHARYNGEAL SWAB COLLECTION INFORMATION** | | | | |
| --- | --- | --- | --- | --- |
| **1.** | Nasopharyngeal swab collected from left nostril | | 1 | Yes  UTM |
|  |  |  | 2 | No 🡪 skip to 1.1 |
|  | 1.1 | If no, please specify the reason::______________________________________ STOP and go to remarks, Form Complete | | |
| **2.** | Sample Collection time *(hh:mm, 24 hr clock)* | | **\|___\|___\|:\|___\|___\|** | |
| **3.** | Swab taken before the ritual cleaning of the left nostril | | Yes  No | |
| **4.** | Sample Storage Condition | | **Cold Packs**   **Wet Ice** | |
| **5.** | Temperature of Coleman at the time of receiving | | - degree C | |

| **FIELD SITE LAB RECEIVING** | | |
| --- | --- | --- |
| **3.** | Name of Person storing the sample | Code: |
| **4.** | Name of Sample depositing Person: | Code: |
| **5.** | Center Receiving Person: | Code: |
| **6.** | Received Time: \|___\|___\|:\|___\|___\| | Received Date : \|___\|___\|/\|___\|___\|/\|___\|___\| |
| **7.** | Temperature of Coleman at the time of receiving | - degree C |

| **Remarks (if any):** |  |
| --- | --- |

**End of Nasopharyngeal swab COLLECTION foRm**
